# Exclusive breastfeeding intention among pregnant women and associated variables: a cross-sectional study in a Brazilian community

**DOI:** 10.1590/1984-0462/2024/42/2022192

**Published:** 2023-10-23

**Authors:** Lorena Fonseca Silva, Karine Laura Cortellazzi, Letícia Santos Alves de Melo, Silvio Rocha Corrêa da Silva, Fernanda Lopez Rosell, Aylton Valsecki, Elaine Pereira da Silva Tagliaferro

**Affiliations:** a Universidade Estadual Paulista, Araraquara, SP, Brazil.; b Universidade Estadual de Campinas, Piracicaba, SP, Brazil.

**Keywords:** Breastfeeding, Intention, Pregnant women, Aleitamento materno, Intenção, Gestantes

## Abstract

**Objective:**

To investigate exclusive breastfeeding (EBF) intention and associated variables among women in the third trimester of pregnancy.

**Methods:**

The data were collected with a questionnaire for the pregnant women (n=653), from December/2018 to November/2019. They answered the Infant Feeding Intentions (IFI) scale, translated and adapted to Brazilian Portuguese, and a questionnaire on sociodemographic, biological, family, pregnancy, breastfeeding, health care, and habits variables. Descriptive statistics and multiple logistic regression analyses were performed with a 5% significance level, following a multilevel hierarchical model that estimated the association between the dependent and independent variables. The outcome EBF intention measured by the IFI score was dichotomized by the median (<16 or =16).

**Results:**

Mean±standard deviation score for the IFI scale was 14.4±2.6 (score 0: very strong intention to not breastfeed at all; score 16: very strong EBF intention up to six months). The results from the regression analysis showed that pregnant women who had no intention to bottle feed (*OR=4.33; 95%CI 2.79-6.72*) or did not know (OR=1.85; 95%CI 1.21–2.82), those who planned the pregnancy (OR=1.52; 95%CI 1.09–2.12), those who believed they would have help to care for the baby (OR=3.60; 95%CI 1.51–8.56) or did not know (OR=3.97; 95%CI 1.26–12.51), and those who reported knowing the World Health Organization recommendations on breastfeeding (OR=1.73; 95%CI 1.13–2.64) were more likely to show a very strong EBF intention.

**Conclusions:**

Pregnant women in the third trimester of pregnancy presented a strong EBF intention. The higher EBF intention score was significantly associated with the structural, setting, and individual determinants.

## RESUMO

**Objetivo:**

Investigar a intenção materna de amamentar exclusivamente (IMA) e variáveis associadas entre as mulheres no terceiro trimestre de gravidez.

**Métodos:**

A coleta de dados foi realizada por meio de entrevista e questionário semiestruturado e autoadministrado, entre dezembro/2018 e novembro/2019. As gestantes (n=653) responderam à escala *Infant Feeding Intentions* (IFI) testada, traduzida e adaptada para o português do Brasil e ao questionário contendo perguntas sobre variáveis sociodemográficas, biológicas e familiares, bem como relacionadas à gestação, ao aleitamento materno, à assistência à saúde e a hábitos. Foram realizadas análises estatísticas descritivas e de regressão logística múltipla hierarquizada, com nível de significância de 5%, para estimar a associação entre as variáveis independentes e o desfecho. A IMA, medida pela escala IFI, foi dicotomizada pela mediana (<16 ou =16).

**Resultados:**

A pontuação média±desvio padrão para a escala IFI foi de 14,4±2,6. Os resultados da análise de regressão mostraram que as gestantes que não tinham intenção de oferecer mamadeira (OR=4,33; IC95% 2,79-6,72) ou não sabiam (OR=1,85; IC95% 1,21–2,82), que planejaram a gestação (OR=1,52; IC95% 1,09–2,12), aquelas que acreditavam que teriam ajuda nos cuidados com o bebê (OR=3,60; IC95% 1,51–8,56) ou que não sabiam (OR=3,97; IC95% 1,26–12,51), bem como aquelas que relataram conhecer as recomendações da Organização Mundial de Saúde sobre amamentação (OR=1,73; IC95% 1.13–2.64) tinham mais chances de mostrar uma IMA muito forte.

**Conclusões:**

As gestantes no terceiro trimestre de gestação apresentaram forte IMA. A maior IMA esteve significativamente associada aos determinantes estruturais, contextuais e individuais.

## INTRODUCTION

Exclusive breastfeeding (EBF) intention is a psychosocial predictor of EBF practices^1^ in women who have given birth.^2,3^ The EBF intention is a strong predictor for success in the initiation^4^ and duration of EBF.^4^ It is a progressively constructed behavior since pregnancy and is influenced by infant and environmental pressures.^5^

The EBF intention among pregnant women has been significantly associated with several factors such as maternal age,^6,7^ maternal education,^6^ knowledge on EBF^7,8^ breastfeeding self-efficacy,^8,9^ parity,^10^ Theory of Planned Behavior,^11^ and gestational body image.^12^

In Brazil, studies have been carried out which assessed breastfeeding intention in postpartum women,^13,14^ a cohort study followed mothers and infants older than three months,^15^ a retrospective survey investigated pregnant women,^16^ and a cross-sectional study followed pregnant women during prenatal care in the public health system.^3^ These studies showed a range from 74.3^15^ to 100%^14^ for the prevalence of breastfeeding intention, thus showing strong breastfeeding intention of the Brazilian women studied. Average breastfeeding durations of 5.5 months for EBF^14^ and 13.5 months for breastfeeding^3^ have been found.

Despite the existence of strong breastfeeding intention among Brazilian mothers, less than 50% of infants under six months old are on EBF.^17^ Considering the strong influence of intention on EBF practices, studies on the understanding of factors associated with EBF intention can help to plan actions and strategies aimed at improving the rates of EBF initiation and duration.

To the best of our knowledge, there is one Brazilian study^18^ that assessed EBF intention with the Infant Feeding Intentions (IFI) scale, to measure the intention to initiate and sustain EBF.^19^ Thus, the present study analyzed EBF intention determined by the IFI scale and associated variables among pregnant women in their third trimester of pregnancy.

## METHOD

This is a cross-sectional study with a convenience sample of pregnant women in the third trimester of pregnancy, that has been approved by a Research Ethics Committee (Certificate of Presentation for Ethical Appreciation — CAAE 96978518.6.0000.5416). All the participants signed an informed consent form before data collection.

This study was performed from December/2018 to November/2019 in a major public maternity hospital, under private rules, with an average of 2132 births per year (45% normal births and 55% cesarean sections) located in a medium-sized city in the center of São Paulo State, Brazil.

The inclusion criteria for participating in the present study were low/high-risk pregnant women in the third trimester assisted by prenatal appointments, emergency care, and ultrasonography. Pregnant women in the third trimester were selected because this is when breastfeeding intentions are usually established.^20^ The exclusion criteria were pregnant women with known contraindications for breastfeeding (mothers infected by HIV, human T-cell lymphotropic virus infection 2 and use of drugs incompatible with breastfeeding), with no response to the outcome variable (EBF intention), who were not Brazilian and who were illiterate.

Six-hundred seventy-eight pregnant women were invited to participate in the survey, of which 655 were accepted and 653 had all data completed for the outcome question. This sample size of 653 participants provided a test power of 0.80 (β=20), a significance level of 5% (α=0.05), for a minimum detectable odds ratio (OR) of 1.8 and 24% response in the unexposed group (considering that 24% of the pregnant women who did not intend to offer a bottle feed had a lower EBF intention). This sample size also agrees with the minimum number of events per variable required in logistic regression analyses.

The data was collected by a single researcher (LFS) supervised by second a researcher (EPST) in the maternity hospital with a semi-structured questionnaire developed based on previous studies on the associated factors for breastfeeding intention^5^ and the literature review conducted to support this study.

Before data collection, a pre-test was conducted at the maternity hospital with 20 pregnant women who met the same selection criteria, to test the methodology and questionnaire comprehension. Pregnant women were asked to report any difficulty in answering the questions. Those with a rate of incomprehension greater than or equal to 20%^21^ and those not filled out by most women were adjusted or removed. The final version of the questionnaire consisted of 72 semi-structured questions. The data collected in the pre-test were not included in the final analysis.

The questionnaire consisted of two parts:

- IFI scale;^19^
- Variables related to sociodemographic, biological, family, pregnancy, breastfeeding, health care, and habits characteristics.

Part 1 was filled out through a structured interview performed by the main researcher (LFS) and part 2 was filled out by the pregnant women. The time spent collecting the data was about 20 minutes per pregnant woman.

The IFI scale was translated and adapted to Brazilian Portuguese^18^ and presented consistency and reliability to assess the EBF intention,^22^ and its use was duly authorized.^19^ The IFI scale was originally developed in English and Spanish versions, showed construct validity and comparability in quantifying maternal breastfeeding intention across multi-ethnic populations,^23^ and represents a simple and valid instrument to assess EBF intention across different contexts.^24^

The IFI scale proposes to quantitatively evaluate the intention to initiate and continue breastfeeding during the first six months of the baby's life. Each item is scored from 0 (very much disagree) to 4 (very much agree), except for item 1 that is scored from 0 (very much agree) to 4 (very much disagree). The first two items assess the intention to initiate breastfeeding and items 3, 4, and 5 assess the intention to offer only breast milk to the baby at one, three, or six months of age.^19^

Total IFI score ranges from 0 to 16, with 0 representing very strong intention to not breastfeed at all and 16 representing very strong intention to offer only breast milk up to six months. The score is calculated by summing the average score of the first two items with the scores of items 3, 4, and 5.^19^

The EBF intention considered EBF with breast milk (milked or straight from the breast) and without any additional food or beverage, except for medication and vitamins, according to the World Health Organization (WHO).^25^

For data analysis, EBF intention (outcome) was dichotomized by the median (<16 or =16). The independent variables (demographic and socioeconomic characteristics, pregnancy, breastfeeding, family, health care, biological, and habits characteristics) were categorized and grouped into four hierarchical levels according to the conceptual model,^26^ considering the determinants of breastfeeding that operate on multiple levels: sociodemographic, structural, setting, and individual.

- Level 1 — Sociodemographic determinants: age (dichotomized by the median at ≤26 or >26 years), years of education (dichotomized by the median at ≤12 or >12 years), and marital status (single/separated or married/stable union).
- Level 2 — Structural determinants: intention to offer a pacifier (yes, no, or unknown), intention to offer a baby bottle (yes, no, or unknown), and “weak milk” belief (yes, no, or unknown).
- Level 3 — Setting determinants: prenatal initiation (dichotomized by the median at ≤2 or >2 months), pregnancy planning (yes or no), belief in having husband/partner support (yes, no, or unknown), living with husband/partner (yes or no), paid job (yes or no), belief in having someone helping to care for the baby (yes, no, or unknown), and the age intended to enroll the baby in daycare or school (up to 1 year or more than 1 year).
- Level 4 — Individual determinants: knowledge of the breastfeeding benefits for the infant (ten benefits with each scoring 1 point, dichotomized by the median at ≤4 or >4 points), knowledge of the breastfeeding benefits for the mother (six benefits with each scoring 1 point, dichotomized by the median at ≤2 or >2 points), knowledge of the WHO recommendations on breastfeeding (yes or no), and previous breastfeeding experience (yes or no).

Hierarchical multiple logistic regression models were estimated. Variables that showed p≤0.20 in the crude analysis were tested in the hierarchical multiple models, remaining in the final models those with p≤0.05. Variables were introduced in the multiple models from the first to the fourth level, with variable adjustments at the same and previous levels. The fit of the models was analyzed by −2 log likelihood. The data were analyzed in the SAS 9.4 statistical software.

## RESULTS

A total of 653 pregnant women participated in this study, providing a response rate of 96.5%.

Tables 1 and 2 presents the descriptive analysis of the variables collected in the sample. Among the women, 39.5% were married, 80.9% lived with their husbands/partners, and 59.6% had no paid work. The age of the pregnant women ranged from 14 to 46 years (mean of 26.8 years) and the years of education ranged from 1 to 26 years (mean of 11.5 years). Most pregnant women reported not having a planned pregnancy (59.7%) and having previous breastfeeding experience (56.8%). Knowledge of the WHO recommendations on breastfeeding was reported by 81.0% of women, 59.6% reported not having a “weak milk” belief, 90.8% believed they would have someone helping to care for the baby, and 86.68% believed they would have husband/partner support. Intention to offer a pacifier or baby bottle was reported by 45.0% and 58.2% of the participants, respectively. The mean score (standard deviation/25^th^–75^th^ percentile) obtained from the IFI scale was 14.4 (2.6/14.0–16.0).

**Table 1:** Descriptive analysis of the categorical variables collected in the sample of pregnant women (n=653).

Variable	Category	Frequency (%)
Marital status	Single	211 (32.3)
	Married	258 (39.5)
	Stable union	166 (25.4)
	Divorced	17 (2.6)
	Not informed	1 (0.2)
Intention to use pacifiers	Yes	294 (45.0)
	No	223 (34.1)
	Undecided	135 (20.7)
	Not informed	1 (0.2)
Intention to bottle feed	Yes	380 (58.2)
	No	151 (23.1)
	Undecided	121 (18.5)
	Not informed	1 (0.2)
Belief in the existence of weak breast milk	Yes	144 (22.1)
	No	389 (59.5)
	Undecided	118 (18.1)
	Not informed	2 (0.3)
Planned pregnancy	Yes	268 (41.0)
	No	385 (59.0)
Husband/partner will provide support	Yes	566 (86.7)
	No	30 (4.5)
	Undecided	56 (8.6)
	Not informed	1 (0.2)
Living with husband/partner	Yes	528 (80.8)
	No	124 (19.0)
	Not informed	1 (0.2)
Paid work	Yes	262 (40.1)
	No	389 (59.6)
	Not informed	2 (0.31)
Someone will help to care for the baby	Yes	593 (90.8)
	No	31 (4.8)
	Not informed	29 (4.4)
Age for enrolling the baby in daycare or school	1–3 months	10 (1.5)
	3–6 months	107 (16.4)
	6–9 months	139 (21.3)
	9 months to 1 year	57 (8.8)
	1 to 1 and a half years	77 (11.8)
	1 and a half to 2 years	42 (6.4)
	2–3 years	73 (11.2)
	After 3 years of age	43 (6.6)
	Undecided	103 (15.7)
	Not informed	2 (0.3)
Knowledge of the WHO recommendations on breastfeeding	Yes	529 (81.0)
	No	123 (18.8)
	Not informed	1 (0.2)
Previous breastfeeding experience	Yes	371 (56.8)
	No	282 (43.2)

WHO: World Health Organization.

**Table 2:** Descriptive analysis of the numerical variables collected in the sample of pregnant women (n=653).

	Mean (minimum-maximum)	Median (25^th^–75^th^ percentile)
Exclusive breastfeeding intention (IFI scale)	14.4 (1.0–16.0)	16.0 (14.0–16.0)
Maternal age	26.8 (14.0–46.0)	26.0 (21.0–31.0)
Years of education	11.5 (1.0–29.0)	12.0 (10.0–12.0)
Knowledge of the breastfeeding benefits for the baby (score)	4.1 (0.0–10.0)	4.0 (2.00–6.00)
Knowledge of the breastfeeding benefits for the mother (score)	2.3 (0.0–6.0)	2.0 (1.00–3.00)
Time of prenatal initiation (months)	2.16 (1.0–7.0)	2.0 (1.00–3.00)

IFI: Infant Feeding Intentions.

Table 3 presents absolute and relative frequencies, mean, and standard deviation for each item of the IFI scale.

**Table 3:** Frequency, mean, and standard deviation for each item of the Infant Feeding Intentions scale among pregnant women (n=653).

Question	Strongly agree	Somewhat agree	Unsure	Somewhat disagree	Strongly disagree	Mean (standard deviation)
	n (%)	n (%)	n (%)	n (%)	n (%)	
1. I am planning to only formula-feed my baby (I will not breastfeed at all)	-	7 (1.1)	19 (2.9)	56 (8.6)	571 (87.4)	3.82 (0.5)
2. I am planning to at least try breastfeeding	616 (94.3)	20 (3.1)	5 (0.8)	8 (1.2)	4 (0.6)	3.89 (0.5)
3. When my baby is 1 month old, I will be breastfeeding without offering any formula or other milk	606 (92.8)	9 (1.4)	17 (2.6)	16 (2.5)	5 (0.8)	3.83 (0.7)
4. When my baby is 3 months old, I will be breastfeeding without offering any formula or other milk	516 (79.0)	61 (9.3)	34 (5.2)	34 (5.2)	8 (1.2)	3.60 (0.9)
5. When my baby is 6 months old, I will be breastfeeding without offering any formula or other milk	365 (55.9)	121 (18.5)	62 (9.5)	71 (10.9)	34 (5.2)	3.09 (1.2)

In the crude analyses, the variables that showed significant association (p≤0.05) with strong EBF intention were higher maternal age, years of education, marital status, intention for pacifier use, intention for bottle feeding, belief in the existence of weak breast milk, time of prenatal initiation, planned pregnancy, belief there will be support from husband/partner, living with husband/partner, belief that someone will help to care for the baby, age intended to enroll the baby in daycare or school, knowledge of the breastfeeding benefits for the mother, and knowledge of the WHO recommendations on breastfeeding (Table 4 and 5).

**Table 4:** Crude analyses of the association of independent variables with Exclusive Breastfeeding intention among pregnant women (n=653) – Levels 1 and 2.

	Variable	Category	Exclusive Breastfeeding intention		Crude OR	95%CI	p-value
			=16*	<16			
			η(%)	η(%)			
Level 1 Sociodemographic determinants	Maternal age	≤26	156 (46.4)	180 (53.6)	Ref		
		>26	183 (57.7)	134 (42.3)	1.58	1.16–2.15	0.004
	Years of education	≤12	278 (49.9)	279 (50.1)	Ref		
		>12	59 (62.8)	35 (37.2)	1.67	1.08–2.65	0.022
	Marital status	Single/ divorced	96 (42.1)	132 (57.9)	Ref		
		Married/ stable union	242 (57.1)	182 (42.9)	1.83	1.32–2.53	<0.001
Level 2 Structural determinants	Intention to use pacifiers	Yes	124 (42.2)	170 (57.2)	Ref		
		No	137 (61.4)	86 (38.6)	2.18	1.53–3.12	<0.001
		Undecided	77 (57.0)	58 (43.0)	1.82	1.21–2.75	0.004
	Intention to bottle-feed	Yes	155 (40.8)	225 (59.2)	Ref		
		No	114 (75.5)	37 (24.5)	4.47	2.93–6.83	<0.001
		Undecided	69 (57.0)	52 (43.0)	1.93	1.27–2.91	0.002
	Belief in the existence of weak breast milk	Yes	66 (45.8)	78 (54.2)	Ref		
		No	217 (55.8)	172 (44.2)	1.49	1.02–2.19	0.042
		Undecided	55 (46.6)	63 (53.4)	1.03	0.63–1.68	0.900

OR: odds ratio; CI: confidence interval; Ref: reference;

* dependent variable reference category (Exclusive Breastfeeding intention).

**Table 5:** Crude analyses of the association of independent variables with Exclusive Breastfeeding intention among pregnant women (n=653) – Levels ¾.

	Variable	Category	Exclusive Breastfeeding intention		Crude OR	95%CI	p-value
			=16*	<16			
			η(%)	η(%)			
Level 3 Setting determinants	Time of prenatal initiation (months)	≤2	249 (54.6)	207 (45.4)	1.43	1.02–2.00	0.037
		>2	90 (45.7)	107 (54.3)	Ref		
	Planned pregnancy	Yes	157 (58.6)	111 (41.4)	1.58	1.15–2.16	0.005
		No	182 (47.27)	203 (52.7)	Ref		
	Husband/partner will provide support	Yes	304 (53.7)	262 (46.3)	2.09	1.18–3.70	0.012
		No	15 (50.0)	15 (50.0)	1.80	0.73–4.43	0.201
		Undecided	20 (35.7)	36 (64.3)	Ref		
	Living with husband/partner	Yes	284 (53.8)	244 (46.2)	1.51	1.02–2.24	0.041
		No	54(43.5)	70 (56.4)	Ref		
	Paid work	Yes	125 (47.7)	137 (52.3)	Ref		
		No	213 (54.8)	176 (45.2)	1.33	0.97–1.82	0.078
	Someone will help to care for the baby	Yes	315 (53.1)	278 (46.9)	3.26	1.43–7.40	0.005
		No	8 (25.8)	23 (74.2)	Ref		
		Undecided	16 (55.2)	13 (44.8)	3.54	1.19–10.50	0.023
	Age for enrolling the baby in daycare or school	Up to 1 year	141 (45.0)	172 (54.9)	Ref		
		More than 1year	135 (57.4)	100 (42.5)	1.65	1.17–2.32	0.004
Level 4 Individual determinants	Knowledge of the breastfeeding benefits for the baby (score)	≤4	182 (50.7)	177 (49.3)	Ref		
		>4	131 (57.2)	98 (42.8)	1.30	0.93–1.82	0.123
	Knowledge of the breastfeeding benefits for the mother (score)	≤2	144 (49.3)	148 (50.7)	Ref		
		>2	116 (60.1)	77 (39.9)	1.55	1.07–2.24	0.020
	Knowledge of the WHO recommendations	Yes	293 (55.4)	236 (44.6)	2.08	1.39–3.11	<0.001
		No	46 (37.4)	77 (62.6)	Ref		
	Previous breastfeeding experience	Yes	201 (54.2)	170 (45.8)	1.23	0.91–1.68	0.184
		No	138 (48.9)	144 (51.1)	Ref		

OR: odds ratio; CI: confidence interval; Ref: reference;

* dependent variable reference category (Exclusive Breastfeeding intention);

Table 6 shows the results of the hierarchical multiple logistic regression, with EBF intention as the outcome. After variable adjustments, the final model showed that pregnant women without intention to offer a baby bottle (OR=4.33; 95% confidence interval — 95%CI 2.79–6.72) or did not know (OR=1.85; 95%CI 1.21–2.82), those who planned the pregnancy (OR=1.52; 95%CI 1.09–2.12), those who believed they would have someone helping to care for the baby (OR=3.60; 95%CI 1.51–8.56) or did not know (OR=3.97; 95%CI 1.26–12.51), and those who reported knowing the WHO recommendations on breastfeeding (OR=1.73; 95%CI 1.13–2.64) were more likely to have strong EBF intention (p≤0.05).

**Table 6:** Hierarchical multiple logistic regression models fitted to describe the influence of independent variables on Exclusive Breastfeeding intention among pregnant women with the variables that showed p-value ≤0.20 in the crude analysis (n=653).

Levels	Category	Model 1			Model 2			Model 3			Model 4 (Final model)		
		OR	95%CI	p-value	OR	95%CI	p-value	OR	95%CI	p-value	OR	95%CI	p-value
Sociodemographic determinants													
Maternal age	≤26	Ref	-	-	-	-	-	-	-	-	-	-	-
	>26	1.44	1.05–1.98	0.025	-	-	-	-	-	-	-	-	-
Marital status	Single/divorced	Ref	-	-	Ref	-	-	Ref	-	-	-	-	-
	Married/stable union	1.70	1.22–2.37	0.002	1.71	1.22–2.40	0.002	1.53	1.07–2.17	0.019	-	-	-
Structural determinants													
Intention to bottle-feed	Yes	-	-	-	Ref	-	-	Ref	-	-	Ref	-	-
	No	-	-	-	4.25	2.77–6.51	<0.001	4.37	2.84–6.74	<0.001	4.33	2.79–6.72	<0.001
	Undecided	-	-	-	1.90	1.25–2.88	0.003	1.87	1.23–2.85	0.005	1.85	1.21–2.82	0.004
Setting determinants													
Planned pregnancy	Yes	-	-	-	-	-		1.41	1.01–1.99	0.048	1.52	1.09–2.12	0.014
	No	-	-	-	-	-		Ref			Ref	-	-
Husband/partner will provide support	Yes	-	-	-	-	-		3.13	1.32–7.44	0.01	3.60	1.51–8.56	0.004
	No	-	-	-	-	-		Ref			Ref	-	-
	Undecided	-	-	-	-	-		3.41	1.09–10.72	0.036	3.97	1.26–12.51	0.018
Individual determinants													
Knowledge of the WHO recommendations	Yes	-	-	-	-	-		-	-	-	1.73	1.13–2.64	0.011
	No	-	-	-	-	-		-	-	-	Ref	-	-

Ref: reference; OR: odds ratio (adjusted); CI: confidence interval; Empty Model (-2log L = 904.293); Final adjusted model (-2log L = 821.851).

## DISCUSSION

This study aimed to contribute to the understanding of factors associated with EBF intention, which is a strong predictor of EBF practices. We used the IFI scale,^19^ which has been translated and adapted to a sample of Brazilian pregnant women.^18^ Among national studies investigating breastfeeding intention,^3,13,14,16^ only two were performed with pregnant women.^3,16^ Moreover, none of these studies used the IFI scale to measure EBF intention, which makes the present study relevant for the subject. The study's findings showed a stronger EBF intention among pregnant women associated with structural, setting, and individual variables.

International studies using the IFI scale have found strong EBF intention. The mean IFI score was 11.80 among urban Hispanic mothers in the United States,^27^ 12.54 among pregnant women in the United Kingdom,^28^ and 13.15 among pregnant women in Slovakia,^12^ indicating slightly lower values than the present study (14.4).

The prevalence of EBF intention for six months among pregnant women varied in the international literature, from 26.7 to 67.0%,^6,9–11^ with the lowest rate in the USA^10^ and the highest in India.^6^ Such differences may arise from cultural, economic, and social differences, such as high-income and low-income countries recording lower and higher EBF intention, respectively. This relationship between EBF intention and socioeconomic differences of countries may reflect the findings on breastfeeding rates of the systematic review by Victora et al.,^2^ which shows a lower prevalence and duration of breastfeeding in high-income countries compared to those with few resources.

There were significant associations among very strong EBF intention (outcome) and planned pregnancy, no intention to offer a baby bottle, belief that someone would help to care for the baby, and knowledge of the WHO recommendations on breastfeeding.

The largest outcome effect size was provided by the variable of no intention to offer a baby bottle (OR=4.33). Pregnant women who felt less comfortable offering infant formula had a higher EBF intention^9^ and those who were more exposed to infant formula advertising during prenatal care were less likely to initiate EBF.^29^ Bottle-feeding may have consequences for the health of the baby, such as the risk of milk contamination and inadequate craniofacial development due to changes in respiratory function, swallowing, chewing, speaking, and dentition.^30^ Furthermore, the use of baby bottles may reduce breast milk production and result in early weaning. Infant formulas, although being proper food for babies, cannot provide all the benefits of breastfeeding.

Pregnant women who reported knowing the WHO recommendations on breastfeeding were more likely to have very strong EBF intention. General knowledge about EBF has also been associated with EBF intention,^7,8^ and it is speculated that the more knowledge on the EBF benefits, the higher the chances of having the desire to initiate and maintain EBF up to the sixth month of the baby's life without offering any other type of beverage or food, as recommended by the WHO.^25^

Pregnant women who believed someone would help to care for the baby were associated with very strong EBF intention. Studies show that the support from the baby's father and/or grandmother is a positive predictor of breastfeeding intention, especially when they are aware of the benefits of this practice and act as stimulators.^10^

There was also an association between very strong EBF intention and planned pregnancy. Studies have shown that women who did not plan their pregnancy were less likely to breastfeed.^5^ These findings suggest the importance of discussing and giving importance to family planning so that pregnancy occurs at the opportune time and women can count on all the necessary assistance before, during, and after this important period.

It is assumed that pregnant women with very strong intention to offer only breast milk for up to six months had prepared to have a baby and acknowledged the importance of EBF and the factors that may harm its establishment. There is arguably a need to strengthen public policies that prioritize family planning and provide pregnant women with opportunities to learn about EBF benefits, to reinforce the intention and act of breastfeeding, and to overcome the potential difficulties of the unique EBF experience.

The limitations of the present study may include its cross-sectional nature, which makes it impossible to trace a causal relationship between independent variables and the outcome; the impossibility of guaranteeing that the absence of associations found is similar to the actual breastfeeding behavior and its practical result; the purely quantitative nature, that did not allow investigating in depth which form of intended feeding, other than breast milk, would be used for the first six months of the baby's life.

The strength of this study lies in the use of a valid scale for quantifying EBF intention and the sample size, with a high response rate.

In conclusion, pregnant women in the third trimester of pregnancy presented a strong EBF intention. The higher EBF intention score was significantly associated with the structural, setting, and individual determinants.

## Data Availability

The database that originated the article is available with the corresponding author.

## References

[1] Lau CY, Lok KY, Tarrant M (2018). Breastfeeding duration and the theory of planned behavior and breastfeeding self-efficacy framework: a systematic review of observational studies. Matern Child Health J.

[2] Victora CG, Bahl R, Barros AJ, França GV, Horton S, Krasevec J (2016). Breastfeeding in the 21st century: epidemiology, mechanisms, and lifelong effect. Lancet.

[3] Fernandes RC, Höfelmann DA (2020). Intenção de amamentar entre gestantes: associação com trabalho, fumo e experiência prévia de amamentação. Ciênc Saúde Coletiva.

[4] Donnan PT, Dalzell J, Symon A, Rauchhaus P, Monteith-Hodge E, Kellett G (2013). Prediction of initiation and cessation of breastfeeding from late pregnancy to 16 weeks: the Feeding Your Baby (FYB) cohort study. BMJ Open.

[5] Vieira TO, Martins CC, Santana GS, Vieira GO, Silva LR (2016). Intenção materna de amamentar: revisão sistemática. Ciênc Saúde Colet.

[6] Behera D, Pillai AK (2016). Intention toward optimal breastfeeding among expecting mothers in Angul district of Odisha, India. Indian J Public Health.

[7] Ihudiebube-Splendor CN, Okafor CB, Anarado AN, Jisieike-Onuigbo NN, Chinweuba AU, Nwaneri AC (2019). Exclusive breastfeeding knowledge, intention to practice and predictors among primiparous women in Enugu South-East, Nigeria. J Pregnancy.

[8] Thomas JS, Yu EA, Tirmizi N, Owais A, Das SK, Rahman S (2015). Maternal knowledge, attitudes and self-efficacy in relation to intention to exclusively breastfeed among pregnant women in rural Bangladesh. Matern Child Health J.

[9] Nommsen-Rivers LA, Chantry CJ, Cohen RJ, Dewey KG (2010). Comfort with the idea of formula feeding helps explain ethnic disparity in breastfeeding intentions among expectant first-time mothers. Breastfeed Med.

[10] Alexander A, O'Riordan MA, Furman L (2010). Do breastfeeding intentions of pregnant inner-city teens and adult women differ?. Breastfeed Med.

[11] Ismail TA, Muda WM, Bakar MI (2014). Intention of pregnant women to exclusively breastfeed their infants: the role of beliefs in the theory of planned behaviour. J Child Health Care.

[12] Mrosková S, Schlosserová A, Reovská M (2018). Analysis of selected determinants of intention to breastfeed. Cent Eur J Nurs Midw.

[13] Oliveira BC, Rodrigues DA, Lamounier JA (2005). Intenção de amamentar e a prática de amamentação em maternidades de Belo Horizonte. Rev Med Minas Gerais.

[14] Machado AK, Elert VW, Pretto AD, Pastore CA (2014). Intenção de amamentar e de introdução de alimentação complementar de puérperas de um Hospital-Escola do sul do Brasil. Ciênc Saúde Coletiva.

[15] Amaral SA, Bielemann RM, Del-Ponte B, Valle NC, Costa CS, Oliveira MS (2020). Intenção de amamentar, duração do aleitamento materno e motivos para o desmame: um estudo de coorte, Pelota, RS, 2014. Epidemiol Serv Saúde.

[16] Moimaz SA, Rocha NB, Garbin CA, Rovida TA, Saliba NA (2017). Factors affecting intention to breastfeed of a group of Brazilian childbearing women. Women Birth.

[17] Universidade Federal do Rio de Janeiro (2020). Estudo nacional de alimentação e nutrição infantil – ENANI-2019: resultados preliminares – indicadores de aleitamento materno no Brasil.

[18] Góes FG, Ledo BC, Santos AS, Pereira-Ávila FM, Silva AC, Christoffel MM (2020). Cultural adaptation of Infant Feeding Intentions Scale (IFI) for pregnant women in Brazil. Rev Bras Enferm.

[19] Nommsen-Rivers LA, Dewey KG (2009). Development and validation of the infant feeding intentions scale. Matern Child Health J.

[20] Rollins NC, Bhandari N, Hajeebhoy N, Horton S, Lutter CK, Martines JC (2016). Why invest, and what it will take to improve breastfeeding practices?. Lancet.

[21] Tamanini JT, D'Ancona CA, Botega NJ, Netto NR (2003). Validação do “King's Health Questionnaire” para o português em mulheres com incontinência urinária. Rev Saúde Pública.

[22] Góes FG, Pereira-Ávila FM, Lucchese I, Ledo BC, Santos AS, Silva AC (2021). Psychometric properties of the brazilian version of the Infant Feeding Intentions Scale. Cienc Cuid Saude.

[23] Nommsen-Rivers LA, Cohen RJ, Chantry CJ, Dewey KG (2010). The infant feeding intentions scale demonstrates construct validity and comparability in quantifying maternal breastfeeding intentions across multiple ethnic groups. Matern Child Nutr.

[24] Yehya N, Tamim H, Shamsedine L, Ayash S, Khalek LA, Ezzi AA (2017). Validation of the Arabic version of the infant feeding intentions scale among Lebanese women. J Hum Lact.

[25] World Health Organization [homepage on the Internet] (2008). Indicators for assessing infant and young child feeding practices: definitions and measurement methods.

[26] Rollins NC, Lutter CK, Bhandari N, Hajeebhoy N, Horton S, Martines JC (2016). Por que investir e o que será necessário para melhorar as práticas de amamentação?. Epidemiol Serv Saéde.

[27] Linares AM, Rayens MK, Dozier A, Wiggins A, Dignan MB (2015). Factors influencing exclusive breastfeeding at 4 months postpartum in a sample of urban Hispanic mothers in Kentucky. J Hum Lact.

[28] Davie P, Bick D, Chilcot J (2019). To what extent does maternal body mass index predict intentions, attitudes, or practices of early infant feeding?. Matern Child Nutr.

[29] Zhang Y, Carlton E, Fein SB (2013). The association of prenatal media marketing exposure recall with breastfeeding intentions, initiation, and duration. J Hum Lact.

[30] Castilho SD, Barros AA, Cocetti M (2010). Evolução histórica dos utensílios empregados para alimentar lactentes não amamentados. Ciênc Saúde Coletiva.

